# Continuing Professional Development—Medical Imaging

**DOI:** 10.1002/jmrs.70134

**Published:** 2026-10-12

Maximise your continuing professional development (CPD) by reading the selected article and answering the five questions. Please remember to self‐claim your CPD and retain your supporting evidence. Answers will be available via the QR code and published in JMRS—Volume 74, Issue 4, December 2027.

## Adherence to Diagnostic Imaging Pathways for Patients Presenting to Emergency Departments With Undifferentiated Acute Abdominal Pain: Impact on Radiation Exposure and Multiple Imaging Testing

Brian Evans, Nicola Giannotti, Ernest Ekpo, https://doi.org/10.1002/jmrs.70097.

1. In the studies examined, how often did doctors follow the recommended Diagnostic Imaging Pathway?
  - 29%
  - 52%
  - 75%
  - 90%
2. Which is a potential consequence of unnecessary imaging?
  - Delayed diagnosis and treatment
  - Improved patient outcomes
  - Reduced healthcare resource use
  - Shorter hospital stays
3. When Diagnostic Imaging Pathways are not followed, how much more likely is a patient to undergo an imaging test involving radiation?
  - Approximately the same
  - Approximately 2 times more likely
  - Approximately 3 times more likely
  - Approximately 5 times more likely
4. A doctor chooses not to consult a Diagnostic Imaging Pathway before requesting imaging. Which factor may explain this?
  - The doctor is unfamiliar with the guideline
  - The doctor perceives that the guideline may limit the imaging tests they can request
  - The doctor's colleagues do not follow the guideline
  - The doctor does not consider checking guideline worthwhile
5. A patient presents with atypical symptoms. How should the Diagnostic Imaging Pathway be used?
  - Follow the guideline exactly, as it will always produce the best outcome
  - Follow the guideline because the benefits always outweigh the risks of deviating from it
  - Use the guideline to inform decisions while considering the individual clinical presentation
  - Avoid using the guideline in atypical presentations

## Answers

Scan this QR code or www.surveymonkey.com/r/JMRS_Dec2026_MI to find the answers.

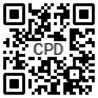
